# The human ovarian surface epithelium is an androgen responsive tissue

**DOI:** 10.1038/sj.bjc.6600154

**Published:** 2002-03-18

**Authors:** R J Edmondson, J M Monaghan, B R Davies

**Affiliations:** Department of Surgery, University of Newcastle upon Tyne, Medical School, Framlington Place, Newcastle upon Tyne NE2 4HH, UK; Northern Gynaecological Oncology Centre, Queen Elizabeth Hospital, Gateshead, Tyne and Wear NE9 6SX, UK

**Keywords:** ovarian surface epithelium, epithelial ovarian cancer, androgens, androgen receptor

## Abstract

The pathogenesis of epithelial ovarian cancer remains unclear. From epidemiological studies raised levels of androgens have been implicated to increase the risk of developing the disease. The purpose of this study was to determine the responses of normal human ovarian surface epithelium to androgens. We have established primary cultures of human ovarian surface epithelium from patients undergoing oophorectomy for benign disease. Total RNA was isolated from these cultures and expression of mRNA encoding for the androgen receptor was demonstrated using reverse transcriptase polymerase chain reaction. The presence of androgen receptor in sections of normal ovary was also investigated using an antibody against androgen receptor. The effects of androgens on DNA synthesis and cell death were determined. Eight out of eight (100%) cultures expressed mRNA encoding the androgen receptor. The presence of androgen receptor in ovarian surface epithelium of sections of normal ovaries was demonstrated in all sections. Mibolerone, a synthetic androgen, caused a significant stimulation of DNA synthesis in 5 out of 9 (55%) cultures when used at a concentration of 1 nM. Mibolerone also caused a significant decrease in cell death in 2 out of 5 (40%) cultures tested. We have demonstrated that the ovarian surface epithelium is an androgen responsive tissue and that androgens can cause an increase in proliferation and a decrease in cell death. These findings have important implications for the pathophysiology of ovarian carcinogenesis.

*British Journal of Cancer* (2002) **86**, 879–885. DOI: 10.1038/sj/bjc/6600154 www.bjcancer.com

© 2002 Cancer Research UK

## 

Ovarian cancer remains a significant health problem. It is a disease which is characterised by late presentation and a poor prognosis with an overall 5 year survival rate of 30%. Approximately 90% of ovarian cancers arise from the ovarian surface epithelium (OSE), a single layer of cuboidal cells which surround the ovary and are in continuity with the peritoneal mesothelium, with which they share a common embryological origin.

The OSE envelopes the ovary and is therefore in close proximity to the underlying stromal, granulosa and thecal cells. These cells are responsible for sex steroid hormone production, namely oestrogen, progesterone and androgens. The factors controlling the proliferation of the OSE are not well understood, but it is highly probable that these high levels of local hormones play some part. Androgens can be postulated to play an important role because of their relatively high levels in the ovary (Risch, 1998). Androstenedione, which is produced by the ovary and the adrenal gland, is a relatively weak androgen but the OSE has been shown to express the enzyme 17β-hydroxysteroid dehydrogenase (Blaustein and Lee, 1979) which can convert androstenedione to testosterone, a much more potent androgen. Androstenedione and testosterone are both found in the developing ovarian follicle, in concentrations exceeding those of oestrogens (McNatty *et al*, 1979; Eden *et al*, 1990). In addition, plasma concentrations of androgens are higher than those of oestrogens, even during the late follicular phase of the cycle (Carr, 1993).

From epidemiological studies, an association between androgens and ovarian cancer has been known for some time. Conditions in which serum levels of androgens are raised such as polycystic ovarian syndrome have been shown to be associated with ovarian cancer (Schildkraut *et al*, 1996), and a nested case control study also showed that elevated levels of circulating androgens were associated with the development of ovarian cancer (Helzlsouer *et al*, 1995). Use of the combined oral contraceptive has been shown to be protective against ovarian cancer (Purdie *et al*, 1995; Vessey and Painter, 1995) and has also been demonstrated to suppress ovarian testosterone production (Gaspard *et al*, 1983). Lastly, administration of testosterone to guinea pigs has been demonstrated to induce benign ovarian epithelial tumours (Silva *et al*, 1997).

Expression of the androgen receptor (AR) has been demonstrated in ovarian cancers using ligand binding assays (Hamilton *et al*, 1981). Further studies have shown AR to be expressed by as many as 90% of epithelial ovarian cancers (Kuhnel *et al*, 1987). There appears to be no correlation between AR expression and histological type of cancer (Chadha *et al*, 1993), and the proportion of cells within each tumour expressing the receptor varies from 10-90% (Chadha *et al*, 1993).

The presence of androgen receptor mRNA (Lau *et al*, 1999) and protein (Chadha *et al*, 1994) has been reported in OSE cells. However, dihydrotestosterone failed to stimulate proliferation of primary cultures of OSE cells (Karlan *et al*, 1995).

In ovarian cancer cell lines it has been shown that administration of dihydrotestosterone results in a dose dependant down regulation of TβR-II mRNA. TβR-II is a receptor for TGFβ, which in turn is a potent inhibitor of epithelial cell proliferation (Evangelou *et al*, 2000). Using primary cultures of ovarian cancers, a dose dependant inhibitory effect of anti-androgens has been demonstrated (Slotman and Rao, 1989). However the administration of dihydrotestosterone at a dose of 100 nM did not lead to an increase in cell number over 10 days (Slotman and Rao 1989).

Several studies have demonstrated an increase in plasma levels of androgens in patients with ovarian cancer when compared with controls (Carlstrom *et al*, 1985; Jeppson *et al*, 1986), and also a correlation between plasma levels of androstenedione with tumour stage and volume (Mahlck *et al*, 1986). Production of androgens is seen to decrease following chemotherapy (Mahlck *et al*, 1986). These reports, taken in conjunction with the data regarding androgen receptor status of tumours, suggest that ovarian cancers have an autocrine growth loop being able to both produce and respond to androgens (Mahlck *et al*, 1986).

Mibolerone is a synthetic androgen (7α,17α-dimethyl 19-nortestosterone) which has several advantages over natural androgens for *in vitro* studies. It is not metabolised, unlike dihydrotestosterone, which can be metabolised *in vitro* (Carlson and Katzanallenbogen, 1990). It binds exclusively to the androgen receptor (Carlson and Katzanallenbogen, 1990) and has been shown to have comparable effects to dihydrotestoterone at doses of 0.1–10 nM (Birrell *et al*, 1995).

In this study we confirm the presence of androgen receptor in hOSE and demonstrate that the synthetic androgen mibolerone can significantly induce cell proliferation and inhibit cell death of primary cultures of normal human OSE.

## MATERIALS AND METHODS

### Materials

Sections of normal ovary were obtained, and primary cultures were established from women undergoing oophorectomy for benign disease, in most cases hysterectomy and oopherectomy for menorrhagia. All patients gave written consent and approval was granted from the local ethics committee.

Primary cultures of human ovarian surface epithelium were established according to the method of Auersperg (Kruk *et al*, 1990). Briefly, ovaries were scraped using a cell scraper to release the ovarian epithelium, which is only tenuously attached to the underlying stroma. The sheets of epithelium removed were transported in Hanks' buffered salt solution (Gibco, Paisley, UK) before centrifugation. They were then resuspended in medium comprising 45% (v/v) RPMI (Gibco, Paisley, UK) and 45% (v/v) Ham's F-12 (Gibco, Paisley, UK) with 10% (v/v) fetal calf serum with added penicillin and streptomycin. Once established the cultures were maintained in this medium in an atmosphere of 5% CO_2_, at 37°C.

Characterisation of the cultures was achieved by staining fixed preparations of the cultures with antibodies to a panel of cytokeratins, in particular cytokeratins 7, 8, 18 and 19 (Auersperg *et al*, 1994). Using costaining techniques, with propidium iodide to stain all nuclei, an estimate of the epithelial content could be made.

The ovarian cancer cell line MDAH 2774 and the androgen sensitive prostate cancer cell lin LNCAP were obtained from the American Tissue Culture Collection (Rockville, MD, USA). The ovarian cancer cell line 41M was obtained from Dr AP Wilson (Oncology Research Centre, Derby, UK).

Mibolerone was obtained from Sigma Chemicals (Poole, Dorset, UK) and reconstituted in phosphate buffered saline.

### Immunohistochemistry

Paraffin sections of formalin fixated tissue were dewaxed in xylene and rehydrated in graded ethanol and treated with 0.5% hydrogen peroxide in methanol for 30 min. Slides were washed in Tris buffered saline before being treated with 0.1 M citric acid pH 6.0 for 20 min at 95°C. Following antigen retrieval the slides were blocked with 20% (v/v) rabbit serum (Dako Ltd, Ely, Cambridgeshire, UK) in phosphate buffered saline (PBS) and 1% (w/v) bovine serum albumin, for 20 min. Sections were incubated at room temperature for 4 h with primary antibody, either clone AR441 mouse monoclonal antibody against androgen receptor (Dako) or clone LP34, mouse monoclonal antibody against pancytokeratin (Dako), both at dilutions of 1 in 50. Slides were washed before being treated with a biotinylated rabbit anti mouse secondary antibody for 45 min, again at a 1 in 50 dilution. The slides were then washed and developed with the streptavidin-biotin/horseradish peroxidase complex kit (Dako) according to the manufacturer's instructions. Finally the slides were dehydrated using graded alcohols before being mounted with DPX.

To act as negative controls sections were also incubated with secondary antibody alone before being processed as described above.

Androgen receptor expression was assessed and scored by two investigators independently (RJ Edmondson and BR Davies). A scoring system was applied with each section being scored with a range of 0–30, calculated as the product of the percentage of cells stained divided by 10 by the intensity of staining (three point scale). Any differences of opinion were resolved by averaging the results.

### RT–PCR

Reverse transcriptase polymerase chain reaction (RT–PCR) was performed on total RNA extracted from primary cultures. 1×10^6^ cells in monolayer culture were trypsinised and centrifuged. Total RNA was extracted using the RNeasy (Qiagen, UK) kit according to the manufacturer's instructions. Total RNA was quantified using a spectrophotometer and 1 μg of RNA was used to construct cDNA using the Superscipt II (Gibco, Paisley, UK) kit, again according to the manufacturer's instructions.

For the androgen receptor an exon spanning fragment stretching from nucleotide position 1653 in exon 1 to position 2845 in exon 4 (Lubhahn *et al*, 1989) was amplified using the sense primer 5′-TGG ATG GAT AGC TAC TCC GG-3′ and the antisense primer 5′-ACT ACA CCT GGC TCA ATG GC-3′ (Nitsche *et al*, 1996), to give a product of 479 base pairs. For GAP-DH expression an exon spanning fragment was amplified using the sense primer 5′- AAA TGA GCC CCA GCC TTC T-3′, and the antisense primer 5′- AGT CAA CGG ATT TGG TCG TA, to give a product of 315 base pairs.

A PCR reaction was made containing sense and antisense primers at a concentration of 200 nM, dNTP mixture at a concentration of 200 μM of each base, 4 units of Taq polymerase and 2 μl of cDNA. For the androgen receptor PCR the mixture was heated to 95°C for 5 min before being subjected to 36 cycles as follows; 94°C for 75 s, 60°C for 90 s and 72°C for 120 s before a final elongation phase at 72°C for 7 min. For expression of GAP-DH the mixture was heated to 95°C for 5 min before being subjected to 22 cycles as follows; 94°C for 60 s, 54°C for 60 s and 72°C for 120 s before a final elongation phase at 72°C for 7 min. In order to calculate the optimum cycle number for every culture, a PCR reaction was performed and stopped after every two cycles. Thirty-six and 22 cycles represent, respectively the linear phase of product accumulation and can therefore be used to semi quantify cDNA concentration.

PCR products were visualised using 2% (w/v) agorose gel electrophoresis and ethidium bromide staining. Gels were photographed on the gel-doc apparatus (Biorad).

A positive control reaction using RNA extracted from the androgen sensitive prostate cancer cell line LNCAP was also performed. A negative control used only buffer in the absence of RNA.

### DNA synthesis assays

DNA synthesis was measured using tritiated thymidine incorporation assay. Cells were plated at a density of 1×10^4 ^cells per well of a 60-well plate. The cells were plated initially in full serum medium. After 4 h this medium was replaced with serum free medium with the addition of bovine serum albumin at a concentration of 250 μg ml^−1^ for a further 24 h in order to reduce the number of actively proliferating cells prior to the addition of growth factors. Varying concentrations of mibolerone were then added to the cultures. Recombinant EGF (1 ng ml^−1^) and IGF (1 ng ml^−1^) were also added to some cultures for comparison. Tritiated thymidine (Amersham, Bucks, UK) was added to the cultures to give a final concentration of 3 μCi ml^−1^ of medium. After 24 h the cells were lifted from monolayer culture with Trypsin/EDTA and lysed with 10% trichloroacetic acid before being harvested onto a glass fibre filter using a cell harvester (Wallac, Turku, Finland). Radioactivity was measured using a scintillation counter (Wallac). All assays were performed using six replicates per treatment group.

### The JAM assay

During cell death one of the most consistent intracellular phenomena is the fragmentation of the genomic DNA. This can be measured using the JAM assay (Matzinger, 1991). Briefly, proliferating cells in monolayer culture were labelled with tritiated thymidine at a concentration of 3 μCi ml^−1^ of medium for 24 h. The cells were then washed to remove unbound tritiated thymidine and cultured in the presence of mibolerone in a 96-well plate. After either 24 or 48 h the cells were lysed and harvested onto a glass fibre mat before being counted on a scintillation counter. During the harvesting process fragmented DNA passes through the glass fibre mat whilst intact DNA is trapped, therefore the amount of radioactivity present on the mat is inversely proportional to the amount of cell death occurring during the time of the assay. Cells exposed to mibolerone were compared to cells in serum free media. Cells were also exposed to staurosporin (Sigma, UK) (2.5 μM), a known inducer of apoptosis as a positive control.

The apoptotic index (AI) is given by the difference between the mean count per minute for the cells with mibolerone and the mean count for cells in serum free medium divided by the mean for cells in serum free medium and expressed as a percentage.

### Statistics

The Mann–Whitney *U*-test was used to compare differences in numbers of androgen receptor positive cells between OSE and epithelium within inclusion cysts. Effects on DNA synthesis and cell death were analysed using the unpaired *t*-test to compare cells stimulated with mibolerone with cells grown in serum free medium. *P* values less than 0.05 were considered significant with *P* values less than 0.01 considered highly significant. The effects of mibolerone across a series of cultures were compared using the two way analysis of variance (two-way ANOVA).

## RESULTS

### Establishing primary cultures of human OSE (hOSE)

**Figure 1:**
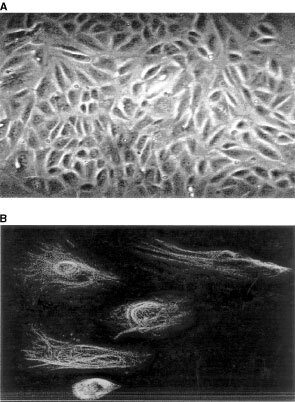
Ovarian surface epithelium in monolayer culture. (**A**) Photomicrograph of normal OSE grown in monolayer culture demonstrating typical appearance of epithelial phenotype. (**B**) Photomicrograph of methanol-fixed OSE cells stained with cytokeratin-18 anti-serum to confirm epithelial phenotype of the cultures.

During the first two passages hOSE cultures were epithelial like in morphology, growing in cobblestone monolayers (Figure 1A

**Figure 4:**
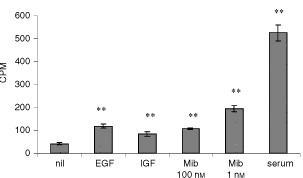
The effects of mibolerone on cells from a primary culture of human ovarian surface epithelium (hOSE 45). Tritiated thymidine was added after the addition of growth factor or 10% serum. Cells were harvested 24 h later. The assay was performed with six replicates. ** Denotes significantly increased DNA synthesis than cells in serum free medium (*P*<0.01 *t*-test).

), but became increasingly elongated as they approached senescence. For this reason, early passage cells with epithelial morphology were utilised in subsequent functional studies. Immediately before carrying out experiments the epithelial phenotype was confirmed using antibodies to cytokeratins (Figure 1B). Cultures utilised for experiments all contained greater than 95% cytokeratin positive epithelial cells.

### Demonstration of the expression of the androgen receptor in normal OSE

**Figure 2:**
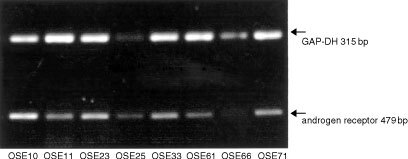
Reverse transcriptase PCR of mRNA extracted from eight primary cultures of normal OSE showing expression of mRNA encoding for androgen receptor compared to expression of the constitutive gene GAP-DH.

A PCR cDNA product corresponding to the androgen receptor mRNA was detected in 8 out of 8 (100%) primary cultures of human OSE (Figure 2

). An identical band was produced using RNA from the androgen sensitive prostate cancer cell line LNCAP, (not shown). The presence of the constitutively expressed mRNA of GAP-DH was confirmed in 8 out of 8 cultures. Expression between cultures was compared using GAP-DH expression as a standard using semi quantitative RT–PCR. The ratio of expression of AR to GAP-DH cDNA product ranged from 0.62 to 1.58 (mean 1.03) indicating that the level of AR expression varied by a factor of 2.5-fold between the highest and lowest expressing cultures. There was no correlation between level of receptor expression and age or menopausal status of the patient from whom the OSE was cultured.

**Figure 3:**
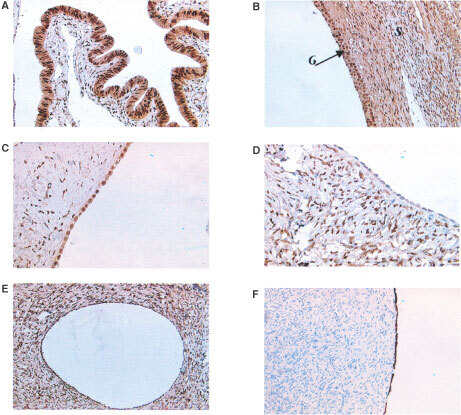
Androgen receptor expression in sections of normal human ovary and fallopian tube. (**A**) Section of normal fallopian tube showing strong epithelial expression of the androgen receptor. (**B**) Androgen receptor positive granulosa (*G*) and stromal (*S*) cells in a developing follicle within normal ovary. (**C**) Androgen receptor positive OSE cells lining a normal ovary. (**D**) Androgen receptor negative OSE cells from a different patient lining a normal ovary. (**E**) Androgen receptor positive epithelium within an inclusion cyst in normal ovary. (**F**) Cytokeratin positive OSE in normal ovary demonstrating epithelial phenotype.

Sections of normal ovary taken from six different patients were examined for the presence of androgen receptor. Immunostaining for AR was strong in adjacent areas of fallopian tube (score 20 out of 30), (Figure 3A

), stromal cells (18 out of 30) (Figure 3B) and granulosa cells (20 out of 30) (Figure 3B). The immunostaining with antisera to the AR in OSE cells was very heterogeneous, with some areas of OSE giving intense positive staining (Figure 3C), whilst other areas remained negative (Figure 3D). However, all normal ovaries contained some AR positive OSE cells (mean score 8.2±2.0). In all cases the OSE examined were situated immediately overlying stromal tissue.

The epithelium within inclusion cysts also stained heterogeneously (Figure 3E), the mean number of AR positive cells was greater than seen in OSE cells (mean 9.7±2.3) but this failed to reach the 5% significance level when the Mann–Whitney test was applied.

Serial sections of ovary were stained with anticytokeratin antisera to aid with identification of epithelial cells (Figure 3F). Negative control sections failed to demonstrate any expression of either androgen receptor or cytokeratin.

### The effect of mibolerone on proliferation of normal OSE cells

The effects of two concentrations of mibolerone on nine primary cultures of human OSE were studied after 24 h incubation. A concentration of 100 nM mibolerone significantly stimulated DNA synthesis in 2 out of 9 primary cultures after 24 h by between 1.6- and 2.6-fold, whilst a concentration of 1 nM caused increased DNA synthesis in 5 out of 9 cultures ranging from 1.3- to 2.8-fold (Table 1

**Table 1:**
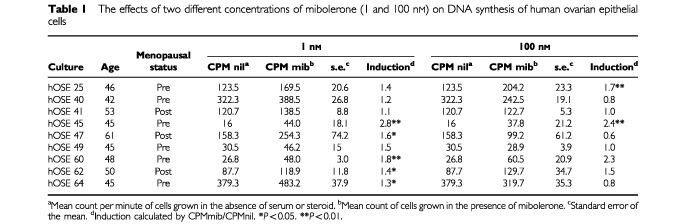
The effects of two different concentrations of mibolerone (1 and 100 nM) on DNA synthesis of human ovarian epithelial cells

). Comparison of means for all cultures tested demonstrates a highly significant increase in DNA synthesis after administration of 1 nM mibolerone, *P*=0.003 (two-way ANOVA), but no significant effect for 100 nM mibolerone, *P*=0.58 (two-way ANOVA).

The magnitude of this stimulation was similar to that of other known growth factors for the OSE, namely EGF (1 ng ml^−1^) (2.8-fold induction) and IGF (1 ng ml^−1^) (2.0-fold induction).

### The effects of mibolerone on proliferation of cancer cell lines

**Figure 5:**
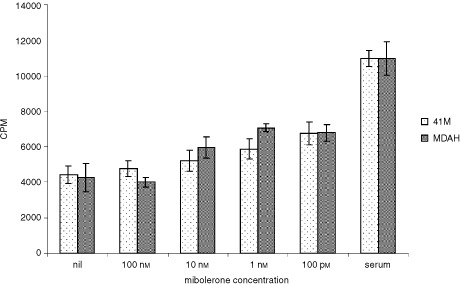
Effect of adding mibolerone to cultures of the ovarian cancer cell lines 41M and MDAH. Tritiated thymidine was added to the cultures immediately following addition of mibolerone and the cells were harvested 24 h later. The increase in DNA synthesis was significantly greater than cells in serum free medium with a dose of 100 pM in both cultures (*P*<0.02, *t*-test).

In the ovarian cancer cell lines 41M and MDAH, concentrations of mibolerone less than 10 nM all slightly induced mean DNA synthesis. This reached statistical significance at 100 pM mibolerone (Figure 5

), which gave a 1.6-fold (*P*<0.02) increase in DNA synthesis when compared with cells in serum free medium.

### The effects of mibolerone on cell death in normal OSE cells

**Figure 6:**
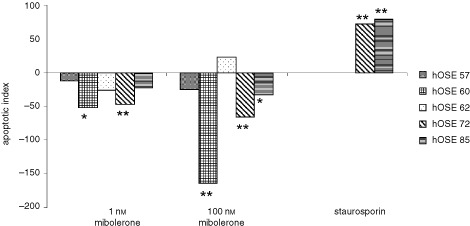
The effect of mibolerone on cell death of OSE cells for five cultures of hOSE exposed to 100 nM and 1 nM mibolerone. The effects of staurosporin, an inducer of apoptosis are shown for comparison. * Denotes significantly more/less cell death than cells in serum free medium *P*<0.05, ** Denotes significantly more/less cell death than cells in serum free medium *P*<0.01.

Mibolerone, at concentrations of both 100 and 1 nM caused a significant decrease in cell death in 3 of 5 primary cultures of hOSE. Cell death was less when cultures were exposed to 1 than 100 nM mibolerone (Figure 6

). As expected staurosporin significantly induced apoptosis (AI 72) (Figure 6).

## DISCUSSION

Although it has long been recognised that the ovary expresses androgen receptors (Al-Timimi *et al*, 1985) this is the first time that the AR has been shown to be functional in human OSE cells. We have demonstrated that in the majority of cases primary cultures of hOSE can respond to androgens within 24 h with a significant induction of DNA synthesis and in some cases a protection from cell death. The use of primary cultures of hOSE allows these cells to be isolated from ovarian influences while at the same time avoiding the adverse cellular changes associated with long-term culture of these particular cells.

The OSE, therefore, is an androgen responsive tissue that plays an integral role in ovarian physiology and is subject to physiological regulation by the androgen synthesising cells of the ovarian follicle and systemic circulating androgens. The factors controlling expression of the AR in human OSE are not known, but appear to be unrelated to age or menopausal status. Given that AR expression is heterogeneous even within individual ovaries, it may be related to the differentiation state of the OSE or controlled by local paracrine factors.

In the physiological state, proliferation of OSE cells is thought to occur during the process of ovulation and/or folliculogenesis. High concentrations of androgens in follicular fluid may play a significant role as paracrine factors in the survival and proliferation of OSE cells. In this context, it is interesting to note that stimulation of DNA synthesis in mouse OSE *in vivo* was reported to be maximal when the OSE was immediately overlying developing follicles (Davies *et al*, 1999).

It is well recognised that the ovarian epithelium forms inclusion cysts and clefts, which are thought to form as part of the ovulatory repair mechanism. It has been suggested that many ovarian cancers arise from these inclusion cysts (Fox, 1993). OSE cells in clefts and cysts also express the AR; and these cells are potentially in closer proximity to the androgen producing cells of the ovarian cortex. This may have relevance in the pathogenesis of EOC.

Previous studies examining the effects of dihydrotestosterone on OSE failed to show any significant increase in DNA synthesis (Karlan *et al*, 1995). The reasons for this difference are unclear, although may be related to different sensitivities of the two assays, cell counting and DNA synthesis, used.

We have shown that androgens can cause a significant stimulation of DNA synthesis within 24 h. It is not known whether the mechanism of stimulation is via induction of cell cycle progression genes such as cyclin D1, androgen-responsive growth factor genes eg FGF-8, or down regulation of the TGFβ-II receptor as in EOC cell lines (Evangelou *et al*, 2000).

Stimulation of the ovarian cancer cell lines with mibolerone leads to a significant increase in proliferation, particularly at the lower doses used. This finding is in contradiction to previous reports which found no increased proliferation in primary cultures of ovarian cancer (Slotman and Rao, 1989), but is consistent with our findings that androgens cause proliferation in OSE, the tissue of origin of ovarian cancer. Since the identification of androgen receptors in ovarian cancers there have been speculations that this represents an autocrine growth loop (Mahlck *et al*, 1986) and a possible target for anti androgen therapy (Rao *et al*, 1990).

In conclusion we have shown, for the first time, that the tissue of origin of epithelial ovarian cancer, the OSE, can respond to androgens. The finding that the normal OSE is responsive to androgens is of major importance in that it opens up the possibility that androgens have a role in ovarian carcinogenesis rather than just represent an upregulated activity within developed malignancy. The physiological mechanisms of androgen action in OSE cells deserve further investigations.
